# Obesity: exploring its connection to brain function through genetic and genomic perspectives

**DOI:** 10.1038/s41380-024-02737-9

**Published:** 2024-09-05

**Authors:** Sadia Saeed, Amélie Bonnefond, Philippe Froguel

**Affiliations:** 1 INSERM UMR 1283, CNRS UMR 8199, European Genomic Institute for Diabetes (EGID), Lille, France; 2 University of Lille, Lille University Hospital, Lille, France; 3 Department of Metabolism, Digestion and Reproduction, Imperial College London, London, UK

**Keywords:** Molecular biology, Genetics, Neuroscience

## Abstract

Obesity represents an escalating global health burden with profound medical and economic impacts. The conventional perspective on obesity revolves around its classification as a “pure” metabolic disorder, marked by an imbalance between calorie consumption and energy expenditure. Present knowledge, however, recognizes the intricate interaction of rare or frequent genetic factors that favor the development of obesity, together with the emergence of neurodevelopmental and mental abnormalities, phenotypes that are modulated by environmental factors such as lifestyle. Thirty years of human genetic research has unveiled >20 genes, causing severe early-onset monogenic obesity and ~1000 loci associated with common polygenic obesity, most of those expressed in the brain, depicting obesity as a neurological and mental condition. Therefore, obesity’s association with brain function should be better recognized. In this context, this review seeks to broaden the current perspective by elucidating the genetic determinants that contribute to both obesity and neurodevelopmental and mental dysfunctions. We conduct a detailed examination of recent genetic findings, correlating them with clinical and behavioral phenotypes associated with obesity. This includes how polygenic obesity, influenced by a myriad of genetic variants, impacts brain regions associated with addiction and reward, differentiating it from monogenic forms. The continuum between non-syndromic and syndromic monogenic obesity, with evidence from neurodevelopmental and cognitive assessments, is also addressed. Current therapeutic approaches that target these genetic mechanisms, yielding improved clinical outcomes and cognitive advantages, are discussed. To sum up, this review corroborates the genetic underpinnings of obesity, affirming its classification as a neurological disorder that may have broader implications for neurodevelopmental and mental conditions. It highlights the promising intersection of genetics, genomics, and neurobiology as a foundation for developing tailored medical approaches to treat obesity and its related neurological aspects.

## Introduction

Obesity, characterized by an excessive accumulation of adipose tissue, has emerged as a major worldwide public health burden together with its associated co-morbidities. The global rise in obesity prevalence with an estimated 4 billion affected by 2035 has prompted extensive research into its underlying causes with the aim for better prevention and more effective medical care [1]. Obesity is a multifactorial disease as both genetic framework and environmental factors contribute to the obesity epidemic. Studies based on twins, families, and adoption data indicate that heritability factors may account for up to 80% of obesity predisposition [2, 3]. However, only less than 10% of severe obesity have been attributed to known genetic causes, so far [4, 5]. This underscores the significant potential of genetic research to identify novel genetic determinants and enhance our understanding of the molecular and physiological pathways regulating body weight. The environmental determinants such as diet, physical activity and stress, overtly influence molecular pathways involved in the energy balance. They interact with genetic variants that modulate individual susceptibility to obesity risk [6]. A relatively new dimension in this context is the field of epigenetics. Here, environmental triggers induce alterations in gene expression without altering the genetic code, acting on the function of the genes but not their structure, positioning epigenetics as an important component in the discourse on obesity [7].

Approximately three decades ago, the discovery of the first human genes involved in monogenic obesity, *i.e.* LEP encoding leptin [8] and *LEPR* encoding leptin ireceptor [9] marked a turning point in our understanding of obesity causes and highlighted the irregularities in the brain function to contribute to this condition. As the genetic basis of obesity expanded with the discovery of additional genes linked to severe, early-onset obesity, the characterization of obesity as a neurological disease has gained clarity. Current knowledge recognizes the brain as essential in regulating energy balance through its orchestration of various functions. This includes the management of neural pathways responsible for appetite and satiety, the modulation of neurotransmitter activity that conveys signals related to energy status, and the integration of neuronal and hormonal signals that inform the brain of the body’s nutritional needs [10].

**Fig. 1:**
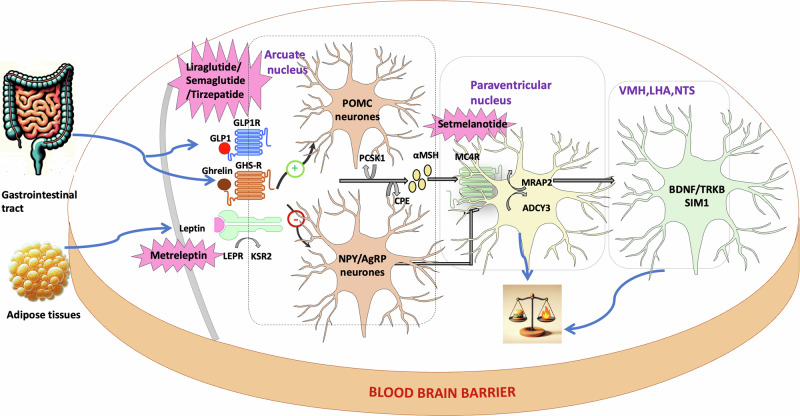
Regulation of energy balance by MC4R signaling in the hypothalamus and associated approved therapeutic agents. ADCY3 adenylate cyclase 3, AgRP agouti-related peptide, BDNF brain derived neurotrophic factor, GLP1 glucagon like peptide 1, GLP1R glucagon like peptide 1 receptor, KSR2 kinase suppressor of ras 2, LHA lateral hypothalamu, MC4R melanocortin-4 receptor, MRAP2 melanocortin 2 receptor accessory Ppn 2, MSH melanocyte-stimulating hormone, NTS nucleus tractus solitarius, PCSK1 proprotein convertase subtilisin/kexin type 1, POMC proopiomelanocortin, SIM1 SIM BHLH transcription factor 1, TRKB tropomyosin-related kinase B, VMH ventromedial nucleus of the hypothalamus.

The central nervous system (CNS), particularly the hypothalamic region, is essential in regulating appetite, satiety, and energy balance. It processes both short and long-term body energy signals, coordinating the secretion of hormones like leptin and ghrelin that modulate hunger (Fig. 1). Neurons in the hypothalamus are closely linked with both neuronal subclusters within this region and with various extrahypothalamic brain regions, enabling a harmonized behavioral response [11].

Available evidence indicates that brain changes causing severe obesity can also lead to significant declines in mental and intellectual abilities. Such alterations adversely affect various cognitive areas, such as decision-making, memory retention, and information processing. In essence, monogenic and polygenic obesity does not just directly impact body weight but can also hinder one’s cognitive performance and overall brain health [12]. However, there are also several secondary mechanisms explaining the strong link between established obesity and CNS abnormalities including sleep apnea, elevated pro-inflammatory cytokines, dysregulated metabolism, oxidative stress, and stigma and discrimination associated with obesity that may lead to stress and mental health issues, including severe depression, eventually affecting cognitive abilities [13].

An understanding of these multiple factors and their interactions provides insights into the complex relationship between obesity and neurological abnormalities, cognitive impairment, and hence the need of a holistic approach to address these issues. The present review focuses on recent progress in the genetics of obesity and seeks to draw attention to the significant yet underappreciated connection between obesity and neurodevelopmental disorders. It advocates for a multifaceted examination of obesity, considering various perspectives to fully understand CNS regulation of energy balance.

## Leptin-melanocortin pathway (LMP) and obesity

Obesity results from an imbalance in the energy metabolism. The hypothalamus is the nexus for maintaining energy equilibrium. It interprets signals from peripheral organs and accordingly fine-tunes our hunger levels [14] through the leptin-melanocortin pathway, the key mechanism for appetite regulation. Any disruption in ligands or receptors involved in melanocortin signaling, such as rare, pathogenic mutations in *LEP, LEPR*, or *MC4R* encoding melanocortin 4 receptor, lead to early-onset severe obesity [4, 15]. Upon its synthesis in adipocytes, leptin undergoes systemic transport to the brain, where it specifically binds to its receptors located within the arcuate nucleus of the hypothalamus. This binding event serves as the triggering point for the activation of the leptin-melanocortin pathway.

Leptin, in this pathway, acts as a stimulator of proopiomelanocortin (POMC) neurons, which are integral in the production of alpha-melanocyte-stimulating hormone (α-MSH). Proprotein convertase subtilisin/kexin type 1 (PCSK1) plays a crucial role in this process by facilitating the cleavage of POMC into its active peptides, including α-MSH. Concurrently, leptin also exerts an inhibitory effect on neurons responsible for generating Agouti-related peptide (AgRP). Notably, AgRP functions as an antagonist to α-MSH, effectively opposing its actions. α-MSH, released from POMC neurons binds to the melanocortin receptor MC4R. This equilibrium between the two neuropeptides, AgRP and POMC is important for maintaining energy homeostasis resulting in a reduction in food intake and an increase in energy expenditure, particularly when energy stores in the form of fat are sufficient [16]. Therefore, both AgRP and POMC neurons exhibit sensitivity to metabolic status [16, 17]. Notably, rare, pathogenic mutations in all the genes involved in leptin driven melanocortin pathway (*LEP*, *LEPR*, *POMC*, *PCSK1*, *MC4R*, primarily [Table 1]) lead to severe early-onset obesity accompanied by eating disorders. These effects are pronounced when mutations occur in a bi-allelic manner for *LEP*, *LEPR*, *PCSK1* and *POMC*, or in a homozygous or heterozygous state for *MC4R*. We have recently shown that total loss-of-function heterozygous mutations in *PCSK1* were also able to cause monogenic obesity [18]. These mutations are commonly associated with additional metabolic and endocrine abnormalities, in addition to severe obesity [5, 19].

**Table 1:** Clinical characteristics of classical monogenic/syndromic obesity.

Gene abbreviation (full name)	Obesity/Hyperphagia	Mode of inheritance	Endocrine/Clinical characteristics	Behavioral/neurological/psychiatrist characteristics
*LEP* (leptin)	Yes/yes	Autosomal recessive	Frequent childhood infections, high childhood mortality, hypogonadotropic hypogonadism, hypothyroidism, thyroid dysfunction, immune dysfunction	Cognitive impairment, slow learning, socialization difficulty, high school dropout rate
*LEPR* (leptin receptor)	Yes/yes	Autosomal recessive	Frequent infections, childhood mortality, hypogonadotropic hypogonadism, hypothyroidism, immune dysfunction, hyperleptinemia	Cognitive impairment, slow learning, socialization difficulty, high school dropout rate
*MC4R* (melanocortin 4 receptor)	Yes/yes	Co-dominant	Hyperinsulinemia, accelerated linear growth,	Mild learning difficulties and behavioral issues, mood disorder, disruptive behavior
*POMC* (proopiomelanocortin)	Yes/yes	Autosomal recessive	ACTH deficiency, altered pigmentation (Red hair, fair skin), hypogonadotropic hypogonadism.	Behavioral abnormalities, possible neurological symptoms, possible decreased pain sensation
*PCSK1* (proprotein convertase subtilisin/Kexin Type 1)	Yes/yes	Autosomal recessive	Hypocortisolism, hypogonadotropic hypogonadism, hyperinsulinemia, malabsorptive diarrhea and other gastrointestinal issues (constipation).	Neurological and developmental issues
*MRAP2* (melanocortin 2 Receptor accessory protein 2)	Yes/yes	Autosomal dominant	Altered adrenal funsion, insulin resistance, dyslipidemia	NA
*SH2B1* (SH2B adaptor protein 1)	Yes/yes	Autosomal dominant	Insulin resistance, dyslipidemia	Impulsivity and attention deficits, emotional dysregulation
*SIM1* (single-minded family BHLH transcription factor 1)	Yes/yes	Autosomal dominant	Altered growth hormone axis, hyperinsulinemia	Behavioral anomalies, cognitive impairment, developmental anomalies
*KSR2* (kinase suppressor of Ras 2)	Yes/yes	Autosomal dominant	Insulin resistance	Learning difficulties
*P4HTM* (prolyl 4-hydroxylase, transmembrane	Yes/yes	Autosomal recessive	Connective tissue abnormalities, hypotonia (muscle weakness)	behavioral or cognitive features, developmental delay, seizures
*ADCY3* (adenylate cyclase 3)	Yes/yes	Autosomal recessive	Anosmia, respiratory complications	Moderate intellectual disability, neurological or neurodevelopmental issues
*ASIP* (agouti-signaling protein)	Yes/yes	Autosomal recessive	Hyperphagia, hyperinsulinemia, overgrowth, hypopigmentation	
*GNAS* (guanine nucleotide binding protein (G Protein), Alpha stimulating activity polypeptide 1)	Yes/yes	Autosomal dominant with imprinting (can vary among different GNAS-related disorders)	Pseudohypoparathyroidism, short stature, round face, subcutaneous calcifications, brachydactyly	Cognitive impairment, development delay, affected motor skills and coordination, seizures
*ALMS1* (Alstrom Syndrome)	Yes/yes	Autosomal recessive	Rod-cone dystrophy, hearing impairment, hypogonadotropic hypogonadism, hypothyroidism, growth hormone deficiency	Developmental delays, intellectual disability or learning difficulties
*BBS1-BBS20* (Bardet-Biedl Syndrome)	Yes/yes	Autosomal recessive	Craniofacial dysmorphism, Polydactyly, hypogonadotropic hypogonadism, genital abnormalities, structural and functional renal abnormalities.	Developmental and cognitive impairments, developmental delay, behavioral problems and poor coordination and balance
*VPS13B* (Cohen Syndrome)	Yes/yes	Autosomal recessive	Retinal dystrophy, progressive myopia, retinochoroidal dystrophy, short stature, delayed puberty.	Developmental delay, intellectual disability, microcephaly, characteristic facial features
*PWS* (Prader-Willi Syndrome)	Yes/yes	Autosomal dominant with imprinting	Growth hormone deficiency, hypogonadism	Cognitive impairment, intellectual disability, behavioral issues (temper outbursts, stubbornness, and compulsive behaviors), sleep disorders, motor milestones delayed

## Leptin-melanocortin pathway (LMP) and eating disorder

Over 30 years of research have demonstrated that mutations in the leptin-melanocortin pathway (LMP) cause various forms of eating disorders. Specifically, homozygous loss-of-function (LoF) mutations in genes such as *LEP*, *LEPR*, and homozygous and heterozygous in *MC4R* result in uncontrollable hyperphagia, an eating disorder characterized by excessive hunger and food intake, leading to extreme early-onset obesity [5, 20].

Surprisingly, some studies have identified a significant link between *MC4R* gain-of-function (GoF) variants and another eating disorder—binge eating disorder (BED)—that may affect obese individuals (Qasim, Mayhew et al. 2019). On the other hand, findings from the UK Biobank (UKBB) general population and another analysis of approximately 17,000 individuals of European origin from nine independent cohorts revealed that carriers of GoF variants in *MC4R* exhibit lower BMI and a reduced risk of metabolic diseases, thereby contributing to a lower prevalence of obesity [21, 22]. These behavioral outcomes are similar to some forms of bulimia (which is often associated with normal weight due to vomiting habits), indicating a complex impact of genetic factors on eating behaviors and their consequences. The differences observed can be attributed to the signaling bias of these variants, where increased β-arrestin recruitment rather than cAMP production results in protective metabolic effects while potentially altering eating behaviors differently [21].

Hypoleptinemia has been shown to trigger various psychological and behavioral adaptations to starvation, which are central to anorexia nervosa (AN) [23, 24]. In AN, reduced leptin levels are a hallmark, critically contributing to the psychological and behavioral manifestations of the disorder implicated in numerous mental health issues, including depression, anxiety, and mood disturbances [25]. Recent study by us highlighted that obese children with genetically driven leptin deficiency exhibit profound behavioral complications including agressive/disruptive behaviors beyond hyperphagia, severely impacting their social interactions and schooling [26]. These children face significant challenges with motivation, reward processing, and stress response due to impaired melanocortin signaling, affecting brain regions outside the hypothalamus. These findings underscore the crucial link between genetic mutations in the LMP and brain circuit dysfunctions, highlighting its significant role in both obesity and opposite eating disorders [23, 24, 27, 28].

## Indirect genetic influences on the LMP and association with obesity

In addition to the main molecular components driving the melanocortin signaling, various modulatory elements and regulatory factors have been elucidated to modify this LMP driven signaling. Indeed, a multitude of signals originating either from the periphery or the CNS are recognized for their roles in modulating feeding behavior and energy expenditure. Melanocortin receptor accessory protein 2 (MRAP2) is responsible for both the trafficking and signaling of MC4R. Loss of MRAP2 function leads to decreased MC4R signaling and obesity in mouse models [29]. Furthermore, monoallelic, pathogenic mutations in *MRAP2* cause monogenic obesity associated with metabolic syndrome in humans [30]. Another example is Adenylate cyclase 3 (ADCY3) that serves as intermediaries for intracellular transmission of the MC4R activation signal [31]. The protein it encodes appears to be co-located with MC4R in the primary cilia of some neurons within the hypothalamus. Furthermore, it has been observed that the suppression of adenylyl cyclase signaling in these cilia leads to an increase in body weight [32]. Biallelic, loss-of-function mutations in *ADCY3* were found to cause severe, early-onset obesity associated with anosmia, cognitive impairment, developmental delay, seizures and severe pneumonia [31]. SH2B Adaptor Protein 1 (SH2B1) acts as a crucial adaptor or scaffolding protein within the LMP and monoallelic, loss-of-function mutations in *SH2B1* could lead to hyperphagia [33]. Initial discovery of this gene’s significance was linked to its presence within a copy number variation (CNVs) on chromosome 16p11.2 among patients with severe early-onset obesity [34].

Furthermore, it has been identified additional genes associated with obesity that influence the LMP, modulating either the development of specific brain regions or the regulation and sensitivity of key components within the pathway (Table 1). For instance, SIM BHLH Transcription Factor 1 (SIM1) plays a role in the development and function of the paraventricular nucleus (PVN) of the hypothalamus. Monoallelic, loss-of-function mutations in *SIM1* were found to be associated with severe, early-onset obesity, possibly associated with a Prader Willi-like syndrome [35, 36]. Therefore, abnormalities in SIM1 can disrupt the normal development and function of the PVN, leading to dysregulation of appetite and energy balance. Kinase Suppressor of Ras 2 (KSR2), modulates the sensitivity of MC4R, and potentially other receptors, to melanocortin hormones. Monoallelic, loss-of-function mutations in *KSR2* were found to be associated with obesity, although the Mendelian mode is still under scrutinity [37]. Agouti Signaling Protein (ASIP) represses MC4R activation and increases food intake. A tandem duplication at *ASIP*, leading to aberrant *ASIP* expression pattern, was found to cause obesity associated with red hair [38]. *GNAS* encodes the stimulatory G-protein alpha subunit protein (Gαs), which mediates G protein-coupled receptor signaling. A recent study showed that monoallelic, loss-of-function mutation of *GNAS* led to obesity by impeding MC4R signaling via defective interaction between Gαs and MC4R [39]. Similarly, recent studies have identified transient receptor potential channel 5 (*TRPC5*) as a significant contributor to obesity and associated behavioral phenotypes by identifying microdeletions on chromosome Xq23 encompassing this gene. TRPC5 is a brain-expressed cation channel that acts on hypothalamic Pomc and oxytocin neurons, playing a crucial role in regulating instinctive behaviors vital for survival, including feeding, arousal, social interaction, and maternal care. Disruption of TRPC5 leads to food seeking behavior, anxiety, autism-like behaviors, and in female mutation carriers, postpartum depression [40].

## Syndromic and non-syndromic obesity: an overlap

Monogenic obesity usually results from pathogenic mutations in a single gene. However, such obesity can also be due to copy-number variations (CNVs) that may span multiple genes, like the 600 kb deletion at chr16p11.2 [41]. Until now, monofactorial obesity has been rigidly classified as syndromic or non-syndromic. The non-syndromic form of monogenic obesity is characterized by the presence of obesity as the sole clinical feature, without the accompaniment of additional symptoms or syndromes. In contrast, syndromic obesity is not only associated with obesity per se, but also with other clinical disorders or features including craniofacial dysmorphism, neurodevelopmental disabilities and different forms/levels of cognitive deficiencies or mental disorders (Table 1). Prominent instances of syndromic obesity, such as Bardet–Biedl syndrome (BBS), exemplify this complexity. BBS is a genetically heterogeneous disorder - caused by biallelic pathogenic mutations in 26 different genes, marked by a set of variable, symptoms/phenotypes including retinal dystrophy, postaxial polydactyly, learning difficulties, hypogonadism, and renal anomalies, alongside obesity [42]. Alström syndrome, caused by biallelic pathogenic mutations in *ALMS1*, encompasses obesity as well as sensory impairments, dilated cardiomyopathy, and metabolic disturbances [43]. Prader-Willi syndrome on the other hand, resulting from aberrations on chromosome 15q11-q13, is characterized by insatiable hunger leading to obesity, intellectual impairment, and endocrine dysfunctions, including growth hormone deficiency and hypogonadism [44].

However, as our understanding of obesity-linked genes and their associated phenotypes expands, these conventional categories become less sharply defined. For instance, monoallelic, loss-of-function mutations in *SH2B1* are associated with obesity and insulin resistance, but they also give rise to neurological and behavioral issues [33]. Patients with a monoallelic, pathogenic *SIM1* mutations often exhibit neurodevelopmental disorders alongside obesity, mirroring the diverse symptoms observed with chromosome 6q deletions [35, 36]. *ADCY3* deficiency is involved in diverse physiological processes and health problems - from anosmia and respiratory problems to learning impairment and metabolic disorders, as mentioned above. Furthermore, *GNAS* deficiency manifests a range of clinical symptoms, including growth, endocrine, and metabolic issues [45]. Patients carrying complete loss-of-function mutations in *P4HTM* have severe hypotonia, cognitive impairment, and developmental anomalies not associated with obesity, although non synonymous mutations cause childhood obesity, also associated with CNS abnormalities [46]. Furthermore, patients with the 600 kb deletion at chr16p11.2, often show autism in addition to fully penetrant obesity, although the reverse duplication of the same region show excessive leanness and schizophrenia [41, 47].

Apart from that, a high mortality or morbidity rates is also observed among the affected children with pathogenic mutations in the LMP. Indeed, the comprehensive clinical evaluations of children deficient for *LEP* or *LEPR* from consanguineous families of Pakistan have unveiled a broader health impact of these mutations beyond obesity. As mentioned above, a significant number of these children also exhibit cognitive and behavioral challenges, including an increased tendency to drop out of school, difficulties integrating with peers (social isolation), and agressive/disruptive behaviors. Moreover, these children exhibited very high rates of life-threatening morbidity and of premature mortality, mainly due to lung or gut infections [26]. This multifaceted impact of leptin signaling deficiency underlines the inadequacy of classifying such conditions as strictly non-syndromic obesity. Instead, these findings highlight a spectrum where a single-gene disorder, traditionally associated with a straightforward phenotype, manifests a range of clinical and cognitive complications, indicating a more complex syndromic-like nature.

## Obesity and brain function

The aforementioned examples of monogenic obesity and associated phenotypes (Table 1) underscore the link between obesity and cognitive/intellectual deficits. Both conditions might be influenced by an overlap of interconnected regulatory pathways thus introducing the concept of shared genetic and epigenetic vulnerabilities. In support of this hypothesis, in a study on consanguineous Pakistani families with childhood obesity, 49% of the probands carried pathogenic point mutations or CNVs in 13 genes/loci responsible for both non-syndromic and syndromic monofactorial obesity in this population. Importantly, the study also identified 28 rare or novel CNVs associated with intellectual disability and/or autism in an additional 22 obese subjects accounting for 10% of cases in this cohort [48].

Damage of brain cells may be secondarily due to an increase in the release of inflammatory cytokines by the adipose tissue that impairs cognitive function over time [49]. Specifically, pro-inflammatory cytokines like interleukin 1 inhibits neuronal activity [13]. This inhibition adversely affects synaptic plasticity and cognitive function. Sleep disorders, such as sleep apnea may also negatively impact cognitive performance. Obesity has also been linked to vascular changes, promoting atherosclerosis. Reduced blood flow to the brain can lead to cognitive decline and an increased risk of neurodegenerative changes. Obesity is associated with chronic low-grade inflammation throughout the body, including the brain. This systemic neuroinflammation may disrupt neural circuits and contribute to cognitive impairments [50]. This chronic low-grade inflammation, can dysregulate the Hypothalamic–pituitary–adrenal axis (HPA axis), impacting cortisol rhythms and potentially contributing to mood disorders, metabolic syndrome, and cognitive impairments. This obesity-induced disruption of the HPA axis, coupled with changes in cortisol secretion, is thought to affect brain structures like the hippocampus, further influencing cognitive function and potentially increasing the risk of neurodegenerative diseases [51].

Neuroimaging studies have revealed structural and functional changes in the brain of individuals with obesity, pointing to potential neural vulnerabilities. The research highlights that childhood obesity has been linked to alterations in brain structure, especially in the prefrontal cortex (PFC) [52], affecting decision-making, response inhibition, working memory, and cognitive flexibility [52]. These findings are in line with changes in gray matter volume, connectivity, and reduced cortical thickness, predominantly in prefrontal regions [53, 54].

## Common polygenic obesity and the neural regulation

**Fig. 2:**
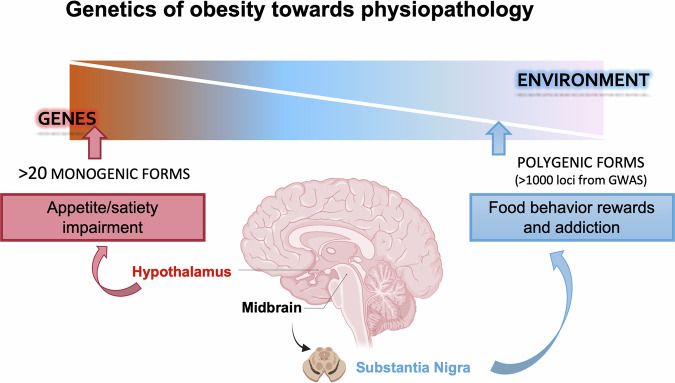
Comparative overview of brain regulation of energy balance in monogenic versus polygenic obesity. Monogenic obesity is characterized by disruptions in the hypothalamic circuits responsible for appetite control, leading to hyperphagia. Contrastingly, polygenic obesity implicates brain regions such as the substantia nigra and the insula, which are associated with addiction and reward processing.

Comprehensive explorations of the human genome via the meta-analysis of 60 genome-wide association studies (GWAS) have identified more than 1,100 independent loci modulating a range of obesity related traits including body mass index (BMI), and increasing risk for common obesity [19, 55]. Importantly, the vast majority of adiposity-associated single nucleotide polymorpisms (SNPs) are located in genes that are expressed in the CNS, bringing the hypothesis that they act on body weight through their impact on brain function. To respond to this question, PCR-free expression assays targeting genes located nearby the GWAS-identified SNPs associated with BMI/obesity were performed in a large panel of human tissues. The study found that the expression of BMI/obesity susceptibility genes was strongly enriched in different regions of the brain, especially in the insula and substantia nigra, which are two brain regions involved in addiction and reward [56]. This pattern of expression of polygenic obesity genes is strikingly different from the one found in monogenic obesity where most of the gene (with the exception of *LEP* expressed in adipocytes) are expressed predominantly in the hypothalamus. Therefore, if both monogenic and polygenic obesity are linked to changes in brain function the mechanisms involved may be different: in monogenic obesity the central circuity of appetite regulation in the hypothalamus is impaired causing hyperphagia, although in polygenic obesity food addiction and other abnormalities in food behavior are involved, with a high sensibility to environment stress (Fig. 2).

Importantly, polygenic susceptibility to obesity can influence monogenic obesity penetrance and the phenotypes of rare variants carriers: for instance, carriers of monoallelic, pathogenic mutations in *MC4R* with a low polygenic risk score (bottom quartile) have an approximately 5-kg/m² lower BMI (approximately 14 kg of body weight for a 1.7-m-tall person) than those with a high PRS (top quartile) [57].

## From genetics to more personalized treatments

Obesity genetic research has led to novel therapeutic options that have revolutionized the conventional lifestyle interventions and are alternative to bariatric surgery that does not work well in most patients with monogenic obesity (in reason of the irrepressible hyperphagia and bigne eating episodes) [58]. Metreleptin, a recombinant leptin analog, has been approved by the FDA for the treatment of congenital leptin deficiency and acquired lipodystrophy. It reduces hunger and body weight among affected individuals and restores normal development and growth in children. In addition, case studies have demonstrated that metreleptin can be effective in treating anorexia nervosa by normalizing low circulating leptin levels. This normalization improves cognitive, emotional, and behavioral effects, alleviating symptoms such as hyperactivity, repetitive thoughts of food, inner restlessness, and weight phobia [23, 59]. Metreleptin can also significantly enhance mood, sleep, and cognitive functions, suggesting that leptin substitution therapy could be a valuable addition to traditional treatments [27]. Setmelanotide, a melanocortin-4 receptor agonist, has shown promising results in treatment of obesity as caused by recessive mutation in *POMC*, *PCSK1*, and *LEPR* deficiencies [60, 61]. Setmelanotide’s effectiveness was also explored in individuals with BBS, yielding noteworthy results in weight loss [62] and decrease in hunger scores.

Glucagon-like peptide-1 (GLP-1) receptor agonists are increasingly used, world-wide in both monogenic [63, 64] and common polygenic obesity [65, 66]. This is not surprising given the physiological effect of GLP-1 on appetite, and the impact of rare variants of *GLP1R* on the variation of BMI [67].

Liraglutide has been approved for the treatment of obesity in adults and adolescents aged 12 years and older. More recently, Semaglutide has demonstrated a higher efficacy compared to other GLP-1 analogs [68].

Emerging strategies, now involve the use of combination drug therapies that target various gut increting signaling mechanisms. Tirzepatide has recently received FDA approval as a treatment for obesity, marking a significant achievement due to its innovative approach of simultaneously targeting GIP and GLP-1 receptors (Fig. 1).

These aformentioned drugs interact with central pathways that regulate appetite and possibly energy expenditure. Moreover, metreleptin, setmelanotide, and liraglutide have been associated with improved cognitive function and mood. This is not surprising as these drugs act on regions of the CNS linked to these brain functions regulation [69]. These findings confirm that obesity is not merely a disorder of energy imbalance but also involves complex neurobehavioral components. It also emphasizes the importance of evaluating the neurobehavioral and mental effects of obesity treatments during the clinical trial process and beyond.

The advent of CRISPR gene-editing technologies holds promise for future treatments that could directly correct the underlying genetic mutations while minimizing adverse effects. The FDA approval of Casgevy, a therapy utilizing the CRISPR–Cas9 gene-editing tool, for sickle-cell disease [70] may open further avenues for monogenic obesity too. The integration of these novel pharmacological agents and technologies into clinical practice signifies a pivotal shift towards precision medicine in the management of obesity. This trend underscores the increasing importance of genetic diagnosis as a prerequisite for selecting the most effective, individualized treatment regimen [71].

## Conclusion

In conclusion, our understanding of obesity as a pure metabolic dysfunction has evolved rapidly, moving away from simplistic notions of ‘calories in’ *vs* ‘calories out’ to a more sophisticated appreciation of the brain’s central role. Obesity appears to be associated with abnormal brain function and mental disorders. Similar to other aspects of obesity, brain abnormalities is an integral part of obesity and needs to be more widely acknowledged in order to facilitate the development of targeted therapies.
